# Label-free SERS assay combined with multivariate spectral data analysis for lamotrigine quantification in human serum

**DOI:** 10.1007/s00604-023-06085-3

**Published:** 2023-12-01

**Authors:** Isidro Badillo-Ramírez, Selina A. J. Janssen, Gohar Soufi, Roman Slipets, Kinga Zór, Anja Boisen

**Affiliations:** 1 Center for Intelligent Drug Delivery and Sensing Using Microcontainers and Nanomechanics (IDUN), Department of Health Technology, Technical University of Denmark, 2800 Kongens Lyngby, Denmark; 2 BioInnovation Institute Foundation, 2200 Copenhagen N, Denmark; 3 Molecular Biosensing for Medical Diagnostics (MBx), Department of Biomedical Engineering, Eindhoven University of Technology, 5600 MB Eindhoven, The Netherlands

**Keywords:** SERS spectroscopy, Lamotrigine, Raman spectrometer, Therapeutic drug monitoring, Antiepileptic drugs, Label-free sensing

## Abstract

Considering the need for a more time and cost-effective method for lamotrigine (LTG) detection in clinics we developed a fast and robust label-free assay based on surface-enhanced Raman scattering (SERS) for LTG quantification from human serum. The optimization and application of the developed assay is presented  showing the: (i) exploration of different methods for LTG separation from human serum; (ii) implementation of a molecular adsorption step on an ordered Au nanopillar SERS substrate; (iii) adaptation of a fast scanning of the SERS substrate, performed with a custom-built compact Raman spectrometer; and (iv) development of LTG quantification methods with univariate and multivariate spectral data analysis. Our results showed, for the first time, the SERS-based characterization of LTG and its label-free identification in human serum. We found that combining a miniaturized solid phase extraction, as sample pre-treatment with the SERS assay, and using a multivariate model is an optimal strategy for LTG quantification in human serum in a linear range from 9.5 to 75 μM, with LoD and LoQ of 3.2 μM and 9.5 μM, respectively, covering the suggested clinical therapeutic window. We also showed that the developed assay allowed for quantifying LTG from human serum in the presence of other drugs, thereby demonstrating the robustness of label-free SERS. The sensing approach and instrumentation can be further automated and integrated in devices that can advance the drug monitoring in real clinical settings.

**Graphical abstract:**

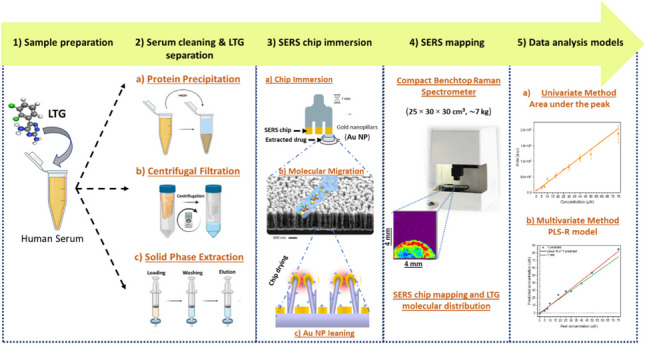

**Supplementary Information:**

The online version contains supplementary material available at 10.1007/s00604-023-06085-3.

## Introduction

Epilepsy is one of the most common severe brain disorders, affecting more than 50 million people worldwide [1]. It is a chronic condition that often lasts for years or even a lifetime, and many patients require long-term therapy with constant antiepileptic drugs (AEDs) administration. Lamotrigine (LTG), 3,5-diamino-6-(2,3-dichlorophenyl)-1,2,4-triazine, is a second-generation AED, which is often prescribed for the treatment of various seizure disorders and maintenance therapy of bipolar depression and psychiatric disorders [2]. The reference range concentration of LTG to treat patients with refractory epilepsy has been suggested to be 3–15 mg L^−1^ (10–59 µM) [3]. Unfortunately, significant inter-patient differences in the dose-to-plasma concentration relationship of LTG are observed because of the pharmacokinetic variability, the type of epilepsy, the severity of the seizures, and the patient’s status [4].

On the other hand, the LTG concentration in the blood can vary due to drug-drug interactions, for example, when coadministered with other AED, like carbamazepine, its half-life is decreased from 24 to 15 h [5]. Also, valproic acid, a frequently administered AED, can prolong LTG concentration up to 60 h [6]. Under the conditions mentioned above, it is clear that implementing therapeutic drug monitoring (TDM) of LTG and other related AEDs is crucial for dose adjustment.

TDM is the common practice of measuring the concentration of drugs in biological fluids at certain time intervals to ensure the effectiveness of the therapy and to prevent the toxicity of the administered drug. The measurement of LTG as part of routine TDM is usually performed in centralized hospital laboratories. Conventionally, these analyses are performed with high-performance liquid chromatography (HPLC) [7], liquid or gas chromatography coupled to mass spectrometry (LC–MS/MS, GC–MS/MS, respectively) [8], or immunoassays [9].

The analytical quantification of LTG and other AEDs in human blood or serum requires the use of bulky and costly instruments (mainly HPLC or LC–MS/MS) that are operated by skilled personnel, implying several time-consuming steps for sample pre-treatment before analysis. These drawbacks result in a waiting time of around 2–3 working days to get a drug concentration value, which implies unnecessary follow-ups for the patients to get a drug adjustment. Therefore, introducing alternative analytical methods that are sensitive, simple to perform, fast (~ less than 1 h), and carried out during a consultancy will improve patient’s care, reduce therapy costs, enable point-of-need TDM, and enhance the routine of drug adjustment.

Surface-enhanced Raman scattering (SERS) has become a promising analytical method for compound quantification purposes in medicine, pharmaceutical, and drug monitoring [10, 11]. However, there is still a need to develop simple and robust assays for real-life applications of SERS, where the sensor can be used with biological samples, such as blood or serum.

In SERS, metallic nanostructured substrates or nanoparticles, mainly silver (Ag) or gold (Au), play a key role in creating an intense local electromagnetic field, which, together with a chemical effect, enhances the vibrational modes of surface-adsorbed molecules of approximately 10^6^ orders of magnitude [12]. The enhanced vibrational modes lead to improved sensitive detection of molecules through their unique Raman fingerprint. Several AEDs relevant for TDM have been investigated with SERS [13, 14]. Nevertheless, to the best of our knowledge, SERS has never been explored to detect LTG.

Although label-free SERS can be used to quantify small organic molecules in simple matrices, its application in complex samples, like biological fluids, is challenging due to the presence of large molecules, such as proteins [15]. Several sample pre-treatment methods have been combined with SERS to reduce serum content interferences, such as protein precipitation, filtration, liquid–liquid extraction, solid–liquid extraction, and solid phase extraction (SPE) [16, 17]. The sample pre-treatment step is often selected to be compatible with the SERS sensor type (e.g., colloids or solid substrates) and the detection approach.

Solid substrates, like ordered Au and Ag nanopillars (Au NP or Ag NP), have been reported in the literature for the sensitive SERS detection of diverse compounds in several matrices [11, 17, 18]. Due to the highly ordered NP structures, they facilitate the separation, migration, and adsorption of small molecules from a complex solution (e.g., biological matrix), unattainable with SERS assays based on metallic colloids [19]. Moreover, compared with colloidal nanoparticles, solid Au or Ag NP substrates are chemically more stable, reproducible, and scalable in manufacturing.

The increase in miniaturized instrumentation has allowed the development of compact Raman spectrometers for on-site SERS analysis [20]. However, in most commercial portable devices, the SERS measurement is performed on single-point acquisition areas, compromising the representative molecular information on the entire SERS substrate, which can decrease measurement reproducibility. Therefore, a modular integration of a benchtop device capable of mapping large areas of the SERS substrate can ensure a quantitative SERS analysis with high reproducibility.

Implementing robust algorithms for drug quantification is highly relevant when aiming to develop analytical methods. Several chemometrics techniques have been used to analyze Raman and SERS data analysis for both classification and quantification, leading to the development of accurate algorithms for drug quantification in complex matrices [17, 21]. Nevertheless, extended applications of SERS for quantitative purposes are still limited, firstly, due to the challenges in manufacturing uniform and reproducible SERS substrates and, secondly, due to the high-intensity variations between individual SERS spot measurements. Some of these drawbacks can be mitigated by addressing substrate uniformity and performing standardized SERS mapping for large dataset acquisition and analysis.

**Fig. 1:**
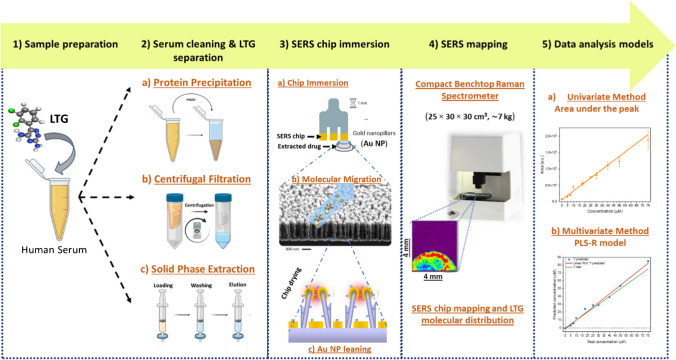
Schematic representation of the experimental steps for the development of the label-free SERS assay for LTG detection and quantification. (1) Sample preparation consisted of spiking LTG in commercial human serum. (2) Protein precipitation, centrifugal filtration, and SPE were tried for serum cleaning and to determine the optimal method for LTG separation. (3) SERS chip immersion procedure consisting of Au NP chip immersion in the clean extracted solution, allowing LTG molecular migration and adsorption after the Au NP leaning. (4) SERS mapping of the entire chip, performed in the in-house built compact Raman spectrometer, allowing to visualize the LTG distribution on the substrate. (5) Spectral data analysis was performed with univariate, area under the peak, and multivariate, the partial least-square regression (PLS-R) model, methods for LTG quantification

In this work, for the first time, we characterized the LTG molecule with SERS and developed a robust and sensitive label-free SERS assay for LTG quantification in human serum. We evaluated several serum sample pre-treatment approaches for efficient LTG separation. We coupled the successful serum cleaning procedures with a straightforward SERS chip immersion method, for analyte migration and adsorption on the ordered Au NP, with immediate SERS chip mapping, performed in a built compact Raman spectrometer. The sensitivity of the SERS assay was improved by implementing univariate and multivariate spectral featured-based calibration models, allowing accurate LTG quantification. Figure 1 shows the general experimental steps for the label-free SERS assay for LTG detection and quantification from human serum spiked with LTG.

## Materials and methods

### Chemicals, solutions, and sample preparation

LTG powder (≥ 98% purity), methotrexate (MTX) powder (≥ 98% purity), imatinib (IMA) powder (≥ 98% purity), and human serum (from human male AB plasma) were purchased from Sigma-Aldrich (Merck, Darmstadt, Germany). Oasis HLB (30 μm), a resin of a copolymer of divinylbenzene (hydrophobic) and vinyl pyrrolidinone (hydrophilic), was purchased from Waters (Milford, MA). The Amicon Ultra 0.5 mL centrifugal filters (3 kDa pore size) were purchased from Millipore-Merck (Darmstadt, Germany). A stock solution of LTG (3 mM) was prepared by dissolving LTG powder in MeOH (HPLC grade), and further desired concentrations were obtained by spiking LTG in commercial human serum or in PBS (pH 7.4). The aliquots were freshly prepared before each measurement. Buffers were prepared with ultrapure water (18.2 MΩ) from a Milli-Q purification system (Millipore Corporation, Billerica, MA, USA). Mixtures of human serum containing LTG and possible interferent drugs that could occur in a clinical setting were prepared as follows: (1) a fresh stock solution of MTX (2 mM) in NaOH (1 M) was spiked in human serum, containing LTG (30 μM), to reach a final concentration of 30 μM; and (2) IMA (1 mM) prepared solution in MeOH was spiked in human serum, containing LTG (30 μM), to a final concentration of 30 μM.

### SERS substrate fabrication

Au SERS NP substrates were prepared by silicon etching followed by electron beam Au metal deposition, as previously reported by Schmidt et al. [22]. See electronic supporting material (ESM) in methods (M1) for a detailed description of the SERS substrate fabrication. Figure S1 shows the SEM image characterization of Au NP after metal deposition. Finally, the Si wafers with the Au NP structures were diced from the backside into ∼4 × 4 mm chips, utilizing a laser micromachining tool (D-09126, 3D-Micromac AG, Chemnitz, Germany). The diced wafers were stored under vacuum in a desiccator until further usage to avoid environmental contamination.

### Compact benchtop Raman spectrometer

All Raman and SERS analyses were performed with an in-house built compact benchtop Raman system (25 × 30 × 30 cm^3^, ∼7 kg) [23]. The Raman device consists of a fully integrated Raman microscope module (Wasatch Photonics, USA), including a multimode laser (785 nm, 450 mW), which yields a laser spot of ∼120 μm size at the sample, allowing a 200–2100 cm^−1^ spectral range, with a 25 μm slit aperture and ∼8 cm^−1^ spectral resolution. The system also includes a motorized XYZ moving stage, based on LT3 (Thorlabs GmbH, Germany), providing a 35 × 35 × 35 mm^3^ scanning volume.

### Raman and SERS characterization of LTG

The reference Raman spectrum was obtained by collecting 10 punctual spectra at random points of the LTG powder, employing 100 mW laser power of a 785 nm laser wavelength and 100 ms of exposure time. Then, an average spectrum was collected after preprocessing with baseline correction.

The reference SERS spectrum of LTG was obtained by dropping 5 µL of LTG solution (25 µM) on the Au SERS chip and letting it dry at room temperature. The entire SERS chip was mapped using a 785 nm laser excitation wavelength, 100 mW laser power, 100 ms of exposure time, and 100 µm in step size. The mapped area was preprocessed for baseline correction, and an average spectrum was obtained in the fingerprint region (400–1800 cm^−1^).

### Serum cleaning and LTG separation

#### Protein precipitation

Protein precipitation of human serum containing LTG (50 µM) was performed by mixing with three different organic solvents (MeOH, EtOH, and ACN) in different proportions (1:1, 1:3, 1:5, and 1:7 v/v of serum to solvent).

#### Centrifugal filtration

Serum samples were filtered using an Amicon Ultra 0.5 mL centrifugal filter unit with a 3 kDa cutoff. The filters were first rinsed with 500 µL of PBS and centrifuged at 10,000 rpm for 30 min (Centrifuge 5430, Eppendorf, Germany). Next, a volume of 500 µL of the human serum containing LTG at different concentrations, and a blank sample, were placed in the centrifugal filter units and centrifuged under the same conditions. The filtered solutions were collected and mixed with MeOH (1:3 ratio) for further analysis with the SERS assay. Each solution was analyzed in triplicates (*n* = 3).

#### Solid phase extraction

The SPE procedure was performed with an in-house developed miniaturized system, a so-called syringe filter holder (μ-SPE-SFH), see ESM in methods (M2) for a detailed description of the fabrication. The commercial serum was spiked with LTG, at the desired concentration, to a final volume of 1 mL and then diluted to 5 mL with Milli-Q water. The diluted serum was loaded in the syringe filter, after preconditioning, following five steps of 1 mL at a flow rate of 1 mL⋅min^−1^. Next, the sorbent was washed with 1 mL of Milli-Q water followed by 1 mL of a mixture of MeOH and Milli-Q water (20:80). Finally, LTG was eluted with 500 μL of MeOH along four cycles. Different numbers of elution cycles (3, 4, 5, and 6) and amounts of MeOH volumes (300, 500, and 700 μL) were evaluated during the optimization of the SPE approach. The schematic representation of the SPE design and separation steps are shown in Figure S2. The collected aliquots were immediately used for analysis with the SERS assay.

##### SERS assay

The SERS-based assay was performed by allowing the LTG molecular migration on the ordered Au NP by controlled immersion of the SERS chip in the sample solution [11]. SERS chip immersion consisted of vertically attaching the SERS substrates (~ 4 × 4 mm), using a double-sided pressure-sensitive adhesive tape, on a fork-shaped holder. Disposable PET reservoirs were used for individual chip immersion. A volume of 50 µL of the sample mixture, containing the analyte and an organic solvent, was placed in the reservoir. Around ¾ of the chip area was immersed in the solution for 1 min, allowing the migration of the solvent and analyte through the NP structures. The SERS chip was removed from the reservoir and dried at room temperature. In the drying step, molecules are adsorbed on the Au metallic surface, and NP structures lean towards each other, creating effective hot spots where a considerable Raman enhancement is expected. Individual SERS maps of the entire chip were collected with the in-house built benchtop Raman system, employing a laser power of 100 mW, a step size of 100 µm, and an exposure time of 100 ms. Each pixel on the map was measured once. A schematic representation of the SERS chip sample immersion and molecular adsorption process, as well as the SERS mapping, is shown in Fig. 1, panels 3 and 4.

##### Data collection and analysis

The acquired SERS maps were analyzed with a custom Matlab code (2018b, MathWorks, MA, USA). Python (Python Software Foundation) and Delphi RAD Studio (Embarcadero Technologies, Austin, TX, USA) were used to develop the Raman data analysis software [23]. A region of 30 × 30 pixels of the acquired map was selected, and a spectral region from 400 to 1800 cm^−1^ was chosen for the band analysis. Baseline correction was performed with a rolling-circle filter (radius of 36 and ellipticity of 38) function. An average spectrum was obtained by selecting the 20% of the pixels that contribute the most to the band at 1356 cm^−1^.

Based on univariate and multivariate spectral data analysis, two approaches were used to build independent calibration plots for LTG quantification, Fig. 1, panel 5. See ESM in methods section (M3) for detailed information of the univariate and multivariate spectral data analysis for LTG quantification.

### Efficiency calculation of serum cleaning methods

The extraction efficiency of LTG was calculated for the centrifugal filtration and SPE methods, see ESM in methods section (M4) for detailed information.

### LTG quantification in complex serum samples with interferent molecules

To show the robustness of the developed SERS assay for LTG quantification even in the presence of interfering compounds, such as anticancer drugs, human serum samples were prepared containing LTG and IMA or MTX as described before. The SPE method was employed for serum cleaning and LTG separation, combined with the SERS assay and SERS mapping analysis. LTG quantification was determined both with the univariate and multivariate spectral methods. Furthermore, the prediction error accuracy of SERS-based quantification was calculated by dividing the predicted concentration by the known concentration multiplied by 100%.

## Results and discussion

### Raman and SERS characterization of LTG

**Fig. 2﻿:**
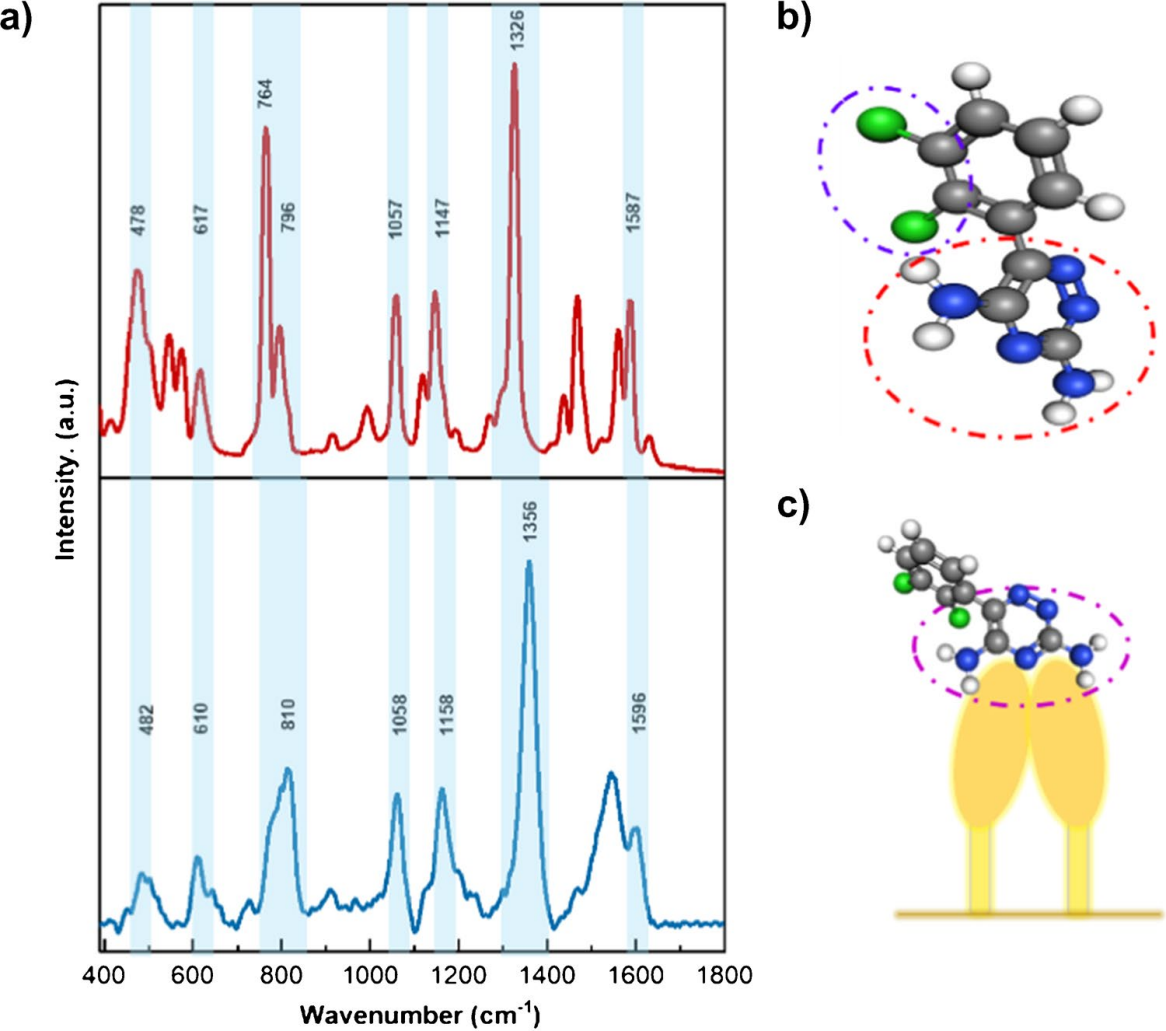
Raman and SERS spectral characterization of LTG. **a** Comparative Raman (red spectrum) and SERS spectra (blue spectrum) of LTG. Highlighted bands and wavenumbers are indicated for related vibrational modes. Detailed band assignment is found in Table S1. **b** LTG molecular structure with indicated functional groups and bonds that are identified in the Raman spectrum fingerprint region, where carbon, nitrogen, hydrogen, and chlorine atoms are indicated with gray, blue, white, and green colors, respectively. **c** Representation of the expected LTG molecular orientation on the Au NP after leaning of the pillars. The pink dotted circle indicates the domain in the molecule that is expected to be enhanced during SERS

Figure 2 shows the comparison between the Raman and SERS spectra of LTG. The Raman spectrum (Fig. 2a, spectrum in red) shows characteristic intense bands of expected functional groups in LTG based on its chemical structure. The most intense band is observed at 1326 cm^−1^, assigned to symmetric and asymmetric C = N stretching modes in the CNN ring [24], as indicated in a red circle in the LTG molecular structure in Fig. 2b. Moreover, intense bands associated with combined modes of CNC stretching and NH_2_ rocking deformations are found at 764 and 796 cm^−1^. Also, in the region 570–610 cm^−1^ characteristic bands of C–Cl stretching, modes are identified [25] and indicated in a purple circle in Fig. 2b. Additionally, medium to intense bands are found at positions 1057, 1147, and 1584 cm^−1^, which are characteristic of aromatic vibrations due to aromatic ring breathing and deformation modes [24, 26]. A detailed Raman band assignment is found in Table S1.

The SERS spectrum of LTG (Fig. 2a, spectrum in blue) shows remarkable characteristic bands that can be correlated with the Raman spectrum, as it is indicated in each wavenumber position and highlighted in light blue in Fig. 2a. For example, the main characteristic band of LTG is identified at 1356 cm^−1^ in the SERS spectrum, which still shows the highest intensity in the fingerprint region. Bands at positions 1158 and 1058 cm^−1^ in the SERS spectrum are attributed to the same vibrational modes as the positions at 1147 and 1057 cm^−1^, respectively, in the Raman spectrum.

It is noticed that some prominent bands identified in the Raman spectrum are shifted and decreased in intensity in the SERS spectrum. For example, bands at 796 and 764 cm^−1^ are overlapped and shifted to 810 cm^−1^ but are attributed to the same vibrational modes. A full SERS band assignment is provided in Table S1.

The identified spectral variations between the Raman and SERS spectra might be attributed to the molecular arrangement and orientation after the deposition on the SERS substrate. In spontaneous Raman, an ordered LTG system and defined intramolecular interactions in the crystal structure are expected to show intense and sharp Raman bands [27]. On the other hand, in the SERS spectrum, when the LTG molecule is in an organic solvent and dried over the Au NP surface, different inter- and intramolecular arrangements might take part after the adsorption on the surface. Also, the molecular LTG orientation over the Au NP might influence the vibrational modes. Functional groups closer to the surface and oriented vertically are expected to have most of their vibrational modes enhanced [28]. In this case, LTG adsorption is anticipated to be most likely via the NH_2_ groups in the aromatic domain, as indicated in a pink circle in Fig. 2c, since a considerably high enhancement of the band at 1356 cm^−1^ is observed [29]. LTG orientation is perpendicular towards the Au NP surface, inducing a high polarizability of the CNN ring close to the substrate, reflected by the intense band at 1356 cm^−1^. A schematic representation of the LTG molecular orientation over the Au NP is shown in Fig. 2c.

### LTG detection in PBS and human serum

**Fig. 3:**
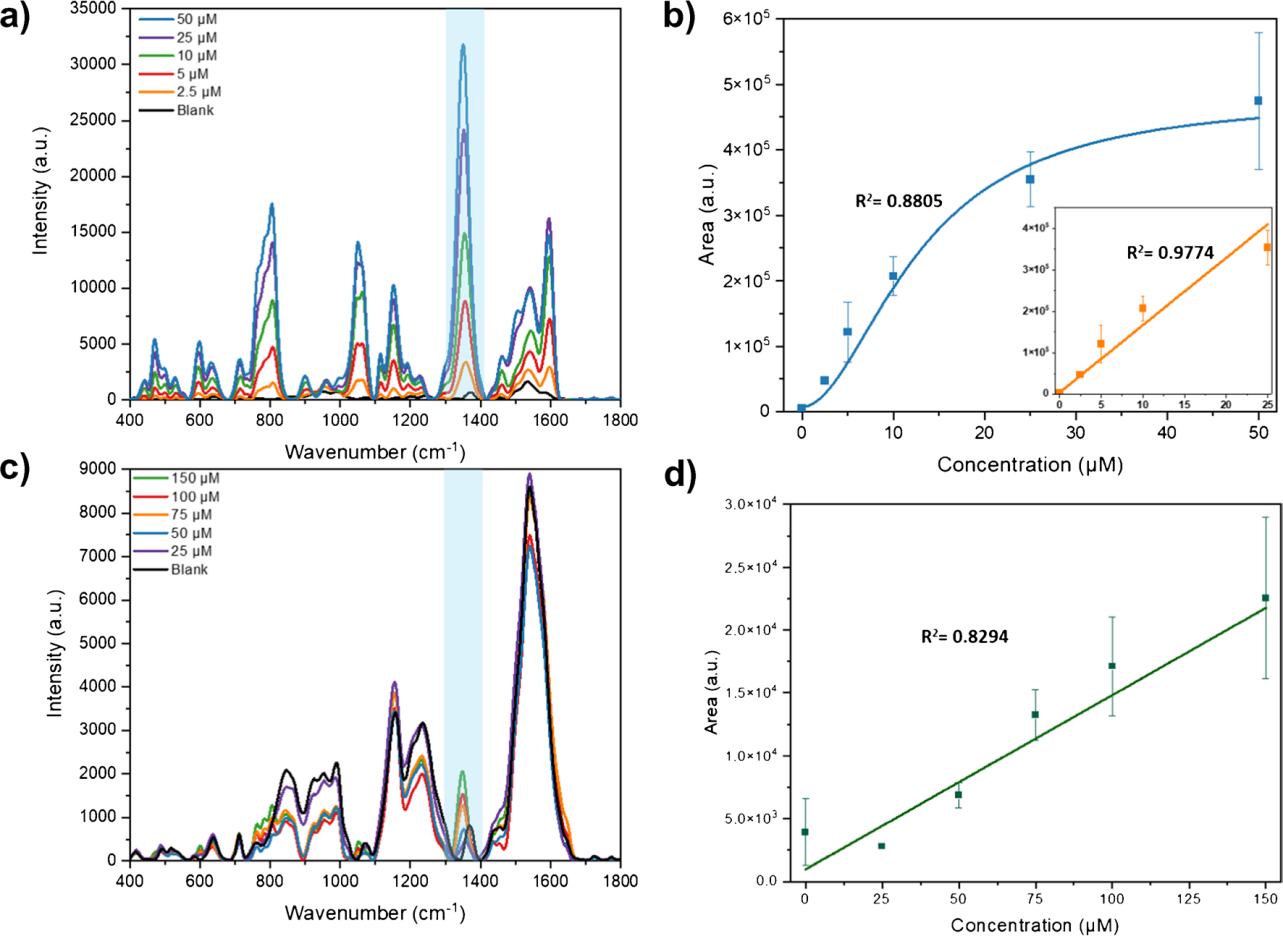
LTG detection in PBS and human serum with SERS. **a** SERS spectra profile of LTG at different concentrations in PBS, with the band at 1356 cm^−1^ highlighted in light blue. **b** Calibration plot with logarithmic curve fitting (sigmoidal fitting Hill equation) of LTG in PBS (average band area ± SD, *n* = 3) with inset showing the linear fitting. **c** SERS spectra profile of LTG in human serum after protein precipitation and SERS-based sensing assay with the band at 1356 cm.^−1^ highlighted in light blue. **d** Calibration plot of LTG in human serum, after protein precipitation, with linear correlation (average band area ± SD, *n* = 3)

The label-free SERS detection of LTG with the SERS chip immersion method was first evaluated by preparing several concentrations of LTG in PBS (pH 7.4). The selection of the organic solvent and mixture ratio was first optimized, considering the ideal conditions based on the high-intensity value of the main LTG band. Figure S3a shows the SERS sensing assay optimization conditions for LTG detection in PBS. Figure 3a shows the LTG spectral features in PBS at concentrations from 2.5 to 50 µM. It is noticed that most of the SERS bands assigned to LTG (Fig. 2a, spectra in red) are still observable. Furthermore, the band at 1356 cm^−1^ (highlighted in light blue) is the most intense of all the cases, suggesting that the LTG molecular orientation on the Au NP is preserved; therefore, this band can be used when building a calibration plot. Figure 3b shows the calibration plot in PBS, built by plotting the mean value of the area under the peak ± SD of three measurements (*n* = 3) of the band at position 1356 cm^−1^ against different LTG concentrations. It is noticed that the main LTG band at 2.5 µM is still distinguishable from the blank solution, and a fitting with a logarithmic curve (sigmoidal Hill equation) is depicted from 2.5 to 50 µM. Similar band area values were observed for concentrations at 25 and 50 µM, which might be attributed to the high enrichment accumulation of LTG on the Au NP, limiting the formation of additional hot spots above 25 µM. Nevertheless, considering the linear range in the calibration plot (2.5 to 25 µM), as is shown in the inset in Fig. 3b, allows us to obtain a correlation coefficient of *R*^2^ = 0.9774. Calculations for the LoD and LoQ values result in 0.20 µM and 1.26 µM, respectively.

As a next step, to show the feasibility of LTG detection in human serum with the SERS-based sensing assay, the protein precipitation method with organic solvents was further investigated as the first strategy of LTG separation in spiked human serum. The organic solvent selection and mix ratio were first optimized as presented in Figure S3b. Figure 3c shows the SERS spectra profile of LTG in serum at different concentrations after protein precipitation with EtOH. It is observed that most of the common LTG SERS bands were obscured by additional broad and intense bands, and only the main band at 1356 cm^−1^ can be noticed, but at low intensity. The change in the SERS band profile and the reduction in band intensity can be attributed to the presence of a high amount of diverse molecules in the serum, mainly to the high abundance of large proteins, that can easily adsorb to the NP structures and foul the surface, limiting the LTG adsorption on the Au NP and obscuring the LTG SERS profile [15].

Several LTG concentrations (25 to 150 µM) spiked in serum were measured, following the protein precipitation method, and a calibration plot was built with the peak area of the band at 1356 cm^−1^ (Fig. 3d). A linear fitting is still observed from 25 to 150 µM. However, the spectral data of concentrations lower than 25 µM are challenging to differentiate from the blank due to high interference of serum molecules with LTG for the adsorption on the Au NP surface. Calculating the LoD and LoQ of LTG in spiked serum led to 78.63 µM and 249.82 µM, respectively. These values are above the recommended clinical reference range (10–59 µM). Therefore, to improve the LTG detection limit in serum, additional strategies for reducing the high serum complexity should be implemented for a label-free SERS assay.

### LTG detection with sample pre-treatment

In a second step, centrifugal filtration and SPE were evaluated for serum cleaning and LTG separation to improve sensitivity detection with the label-free SERS assay.

#### Effect of centrifugal filtration

The centrifugal filtration method is a straightforward strategy to clean up complex biological samples and separate small molecules that can pass through a defined filter pore size.

Figure S4a shows the SERS profile of serum containing LTG [50 µM] after centrifugal filtration, comparatively with the SERS profile after protein precipitation at the same concentration. In a general inspection, it is observed that some bandwidths and intensities are slightly changed after centrifugal filtration. For example, the bandwidth shrinkage and reduced band intensity at around 1550 cm^−1^ reflect the decrease of the protein content in the serum after the centrifugal filtration. On the other hand, some bands increase in intensity after the filtration, like the band at 639 cm^−1^, which is attributed to uric acid, meaning that the 3 kDa filter pore also allowed to pass small endogenous molecules present in the serum. It is evident that the band at 1356 cm^−1^ also shows an increase in intensity, [16, 30] indicating that LTG can effectively pass the 3 kDa filter pore. Therefore, a calibration plot was built (Figure S4b), following the area under the peak method at different LTG concentrations, obtaining a linear range between 25 and 75 µM, and calculated LoD and LoQ of 24.93 µM and 103.85 µM, respectively. Although the centrifugal filtration method improved the LTG sensitivity detection, the obtained values are still above the suggested clinical reference range. The reduced improvement in sensitivity can be attributed to the presence of high amounts of small endogenous molecules that also passed the filter and competed with LTG for the surface adsorption. Therefore, the introduction of a more efficient serum clean-up method might allow the application of label-free SERS for a quick assay for LTG quantification in clinical range concentrations.

#### Effect of SPE

**Fig. 4:**
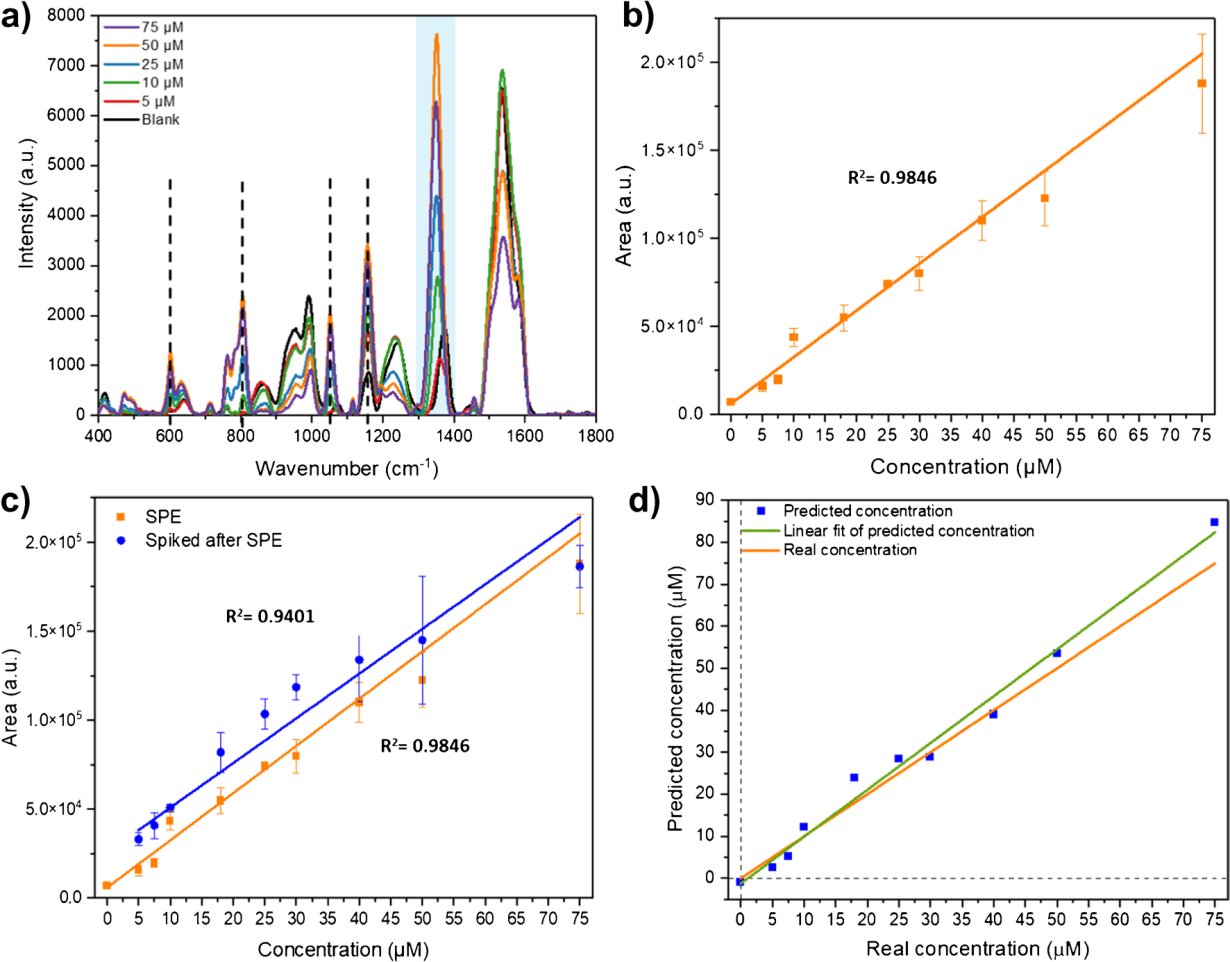
LTG separation with SPE coupled to the SERS-based sensing assay detection method in the compact benchtop Raman. **a** Comparative SERS spectra profile of LTG in serum at different concentrations, where dotted lines indicate the additional observed LTG characteristic vibrational bands. **b** Calibration plot (average band area ± SD, *n* = 3) of the area under the peak of the band at 1356 cm.^−1^ after SPE at different LTG concentrations from 5 to 75 µM. **c** Estimation of the SPE procedure efficiency by comparing the calibration plot (average band area ± SD, *n* = 3) of LTG after SPE (orange line) and the calibration plot of LTG spiked to serum after SPE (blue line). **d** Prediction of the LTG concentration by the PLS-R model based on the SERS spectral features in the fingerprint region; the blue squares are the values predicted by the model per concentration, fitted in a linear equation (orange line), while the green line represents the predicted concentration, *n* = 3

To improve the LTG detection sensitivity, the μ-SPE-SFH method was first optimized and then combined with the SERS-based sensing assay. Figure S5 shows the optimization parameters for LTG separation, based on the selection of optimal amount of volume of the desorption solvent and number of desorption cycles. The obtained SERS spectra profiles of LTG at different concentrations in serum after SPE are visualized in Fig. 4a. The bands related to serum components show less interference, while the additional characteristic LTG bands are better defined, and the main band at 1356 cm^−1^ is of higher intensity, leading to better band identification compared to the centrifugal filtration (Figure S4) and the protein precipitation (Fig. 3c) methods. The collected spectra also allow observing additional characteristic LTG bands, such as 1158 and 1058 cm^−1^, dotted lines in Fig. 4a, that were obscured in the previous sample pre-treatment approaches. Also, the bands at 810 and 610 cm^−1^ are better defined and increased in intensity.

#### Univariate LTG quantification

The measured LTG concentrations after SPE were plotted against the corresponding value of the area under the peak of the band at 1356 cm^−1^, and two correlation models were used to build a calibration plot. A sigmoidal curve (Hill model) fits in the range of concentration from 5 to 100 µM (Figure S6), with a linear correlation found from 5 to 75 µM (Fig. 4b) and calculated LoD and LoQ of 1.82 µM and 11.30 µM, respectively. These values are considerably improved compared to the previous sample pre-treatment methods, showing the benefit of SPE, leading to cover the suggested LTG therapeutic reference range in clinics.

Furthermore, the EE of the SPE procedure was determined by comparing the calibration plot of LTG constructed with samples where LTG was added to serum before SPE (orange line) to the plot where LTG was spiked to serum after SPE (blue line) (Fig. 4c). The LTG concentration of SPE serum spiked with LTG is considered the actual concentration. Since the starting volume of the serum samples was 1 mL, and the elution volume was 500 μL, equivalent to a two times up-concentration, the band intensity of the actual LTG concentration is, thus, expected to be twice the value of the one after SPE. The obtained orange and blue curves are approximately the same. Therefore, the EE of the SPE procedure was found to be 39.9 ± 6.1% (15% RSD), (average value ± SD, *n* = 3), by only considering concentrations above the LoQ (10 μM and higher). The obtained value is comparable to EEs reported for different types of aromatic molecules, employing Oasis HLB, ranging from 40% to around 80% [31].

#### Multivariate LTG quantification

On the other hand, to improve the LTG quantification after SPE and the SERS-based sensing assay, a multivariate method, the PLS-R model, was built considering most of the SERS spectral features of LTG in the region from 500 to 1400 cm^−1^. Several concentration points were collected between 5 and 75 μM. Figure 4d shows the PLS-R predictive model for LTG concentration (blue squares) against the real measured concentrations (orange line), and the PLS-R model parameters employed for the prediction are described in Table 1. The correlation is fitted by a linear equation, green line (*Y* = *XB* + *E*, where *B* is the matrix of regression coefficients and *E* a matrix of residuals) in the range of 5 to 75 μM, with calculated LoD and LoQ of 3.2 μM and 9.5 μM, respectively. These values were improved compared to the results obtained by the area under the peak model. Indeed, including most of the SERS spectral features in the PLS-R model provides a more accurate method for LTG quantification, enabling coverage of the suggested therapeutic range.

**Table 1:** PLS-R details employed for the linear regression model

Figures of merit	Parameter	Value
Accuracy	RMSEC	4.21 µM
	RMSECV	4.88 µM
	RMSEP	4.18 µM
Systematic error	BIAS	− 0.13 µM
Precision	SEP	0.61 µM
Sensitivity	SEN	90.1
Analytical sensitivity	ɣ	1.96 µM^−1^
Inverse of analytical sensitivity	ɣ^−1^	0.51 µM
Fitting	*R*^2^	0.9646
	*R*^2^ CV	0.9530
	*R*^2^ prediction	0.9874

Different types of cross-validation methods (random samples w/10 splits and leave one out) were utilized, along with distinct datasets for predictive analysis, to assess the model’s accuracy. The results, presented in Table S2 and Figure S7, indicate that there are no notable distinctions between RMSECV and R-squared cross-validation (*R*^2^ CV) when applying any of these cross-validation methods. Furthermore, when optimizing with different prediction sets, including those with concentrations not replicated in the training set, there were no significant alterations observed in the RMSEP and the R-squared prediction (*R*^2^ prediction).

Although the PLS-R model was only trained with the SERS spectral data of two measurements per concentration, the predicted concentrations correlate well with the actual measured values (Table S3). Improved concentration prediction can be obtained with a larger set of spectral data in the PLS-R model.

### LTG quantification in complex samples with interferent drugs

To show the efficiency of the optimized SPE procedure combined with the SERS sensing assay, as well as the interference effect of other TDM drugs on the quantification models, LTG was prepared in serum samples with the addition of MTX and IMA as interferent drugs. These drugs were selected because (i) firstly, in clinical epileptic patients that converge with a cancer treatment, it is usual that MTX or IMA, commonly prescribed oncological drugs, which are coadministered with LTG, and, in some cases, an individual TDM assay is required for each drug; (ii) secondly, to test the robustness of our assay and to detect molecules that are able to be identified with the same SERS substrate, we previously identify that these two molecules are SERS active on the same Au NP substrate [11, 32].

Figure S8 shows the acquired average SERS spectra of the complex serum samples in the fingerprint region. In a first inspection, it is noticed that in samples 1 and 2, the characteristic SERS bands of LTG are identified with variations in band intensities, suggesting different LTG concentrations in the complex matrix. On the other hand, extra SERS band features are observed for samples 3 and 4, as expected from the presence of the spiked oncological drugs in the serum.

The presence of MTX is observed as a shoulder of low intensity around 689 cm^−1^. This molecule has been characterized previously with SERS in serum by employing different SERS type substrates [11, 17]. The spectrum of sample 4 shows a clear additional band at ~ 1034 cm^−1^, characteristic of IMA, that has been characterized previously with Raman and SERS in human plasma [32, 33].

The performances of the two calibration models, i.e., the area under the peak and PLS-R, were evaluated for LTG quantification in the complex samples. The obtained concentration values and the real sample composition and concentrations are listed in Table 2.

**Table 2:** LTG quantification in serum samples in the presence of interfering compounds and predicted values by comparing the two developed quantification methods

Sample	Real concentration and sample composition	Predicted LTG concentration with the peak area model [µM]	Recovery (%)	Predicted LTG concentration with the PLS-R model [µM]	Recovery (%)
1 (*n* = 3)	LTG [30 µM]	23.3 ± 1.3	77.7 ± 4.4	27.5 ± 2.5	91.5 ± 8.2
2 (*n* = 4)	LTG [15 µM]	17.0 ± 4.3	113.6 ± 30.3	20.3 ± 4.9	135.3 ± 32.6
3 (*n* = 3)	LTG [30 µM] + MTX [30 µM]	27.0 ± 5.4	90.1 ± 18.0	30.9 ± 3.2	102.3 ± 10.6
4 (*n* = 4)	LTG [30 µM] + IMA [30 µM]	10.3 ± 2.6	34.4 ± 8.8	19.9 ± 4.1	66.4 ± 13.6

The concentration of samples 1 and 2, containing only LTG, can be predicted with both methods, where the PLS-R model is more accurate for the 30 µM and the peak area method for the 15 µM concentration. On the other hand, LTG concentrations in samples 3 and 4 are predicted with higher accuracy with the PLS-R model. The lower predicted accuracy for LTG in sample 4 is attributed to the presence of IMA, which shows a characteristic band (1034 cm^−1^) closer to a LTG band at 1058 cm^−1^, which in consequence lowered the predicted LTG concentration employing the PLS-R model. The noticeable presence of IMA in the spectrum can be originated from the co-elution of IMA and LTG after the SPE method and their possible molecular competition to the NP surface, reducing the number of LTG molecules adsorbed onto the Au NP. The factors that decrease the LTG prediction accuracy in both methods are the band overlapping and the decrement in band intensities. Nevertheless, the multivariate analysis shows a better accuracy prediction for LTG quantification, compared to the univariate spectral analysis, even in high complex samples.

### Comparison of the SERS assay with other LTG detection methods

Table 3 presents the characteristics of several published detection methods and sensors, where LTG has been measured in various sample types, compared with the here-developed assay, without including methods based on standard liquid chromatography and immunoassays.

**Table 3:** Comparison of our SERS assay with other reported methods for detecting LTG

Technique	Sample matrix	LTG separation	Linear range	LoD	LoQ	Ref
Digital colorimetric assay	Exhaled breath condensate	Sucrose-functionalized Au nanoparticles	0.39–27.33 μM	0.16 μM		[34]
Cyclic voltammetry	Pharmaceutical and plasma	Label-free	1–100 μM	80 nM		[35]
Spectrophotometry	Plasma	Emulsification and microextraction	1.95–39.4 μM	1.7 μM		[36]
Differential pulse voltammetry	Urine and plasma	Magnetic MIPs-based extraction	0.01–1.0 nM and 1.0–200 nM	4.7 pM urine; 5.9 pM plasma		[37]
Fluorescence	Plasma	FRET probes	1.90–23.42 µM	1.17 µM		[38]
Fluorescence	Tablets, urine, and plasma	Magnetic graphene oxide nanocomposite	7.8–175.7 nM	1.5 nM	4.92 nM	[39]
SERS	Serum	Label-free	9.5–75 μM	3.2 μM	9.5 μM	This work

These results show that the SERS assay developed in this paper is comparable with several published methods [36, 38] especially when LTG was detected in the biological sample. Although there are reported techniques for LTG with very high sensitivity [37, 39], these methods require the use of additional steps, such as the formation of ion-pair, charge-transfer complex compounds, or molecular imprinted polymers, involving extra time for preparation and the use of expensive reagents. On the other hand, reported label-free methods for LTG detection in biological samples emphasize the decrease in sensitivity due to the surface fouling effect of large molecules from the matrix towards the surface of the sensing element [35, 38].

The interference and competition of molecules with similar chemical structure to our target analyte on the sensing substrate is one limitation identified in our assay, which is always a big challenge for the development of label-free SERS approaches for molecular quantification from biological samples. This can lead to a decrease in sensitivity and lower accuracy in detection. However, these challenges can be addressed by making more robust analytical prediction models (advanced multivariate analysis approaches). Nevertheless, it is important to mention that there is no need to quantify LTG in extremely low concentrations in clinical practice, considering that the suggested clinical reference range is 10–59 µM [3]. In this sense, the here-presented method shows that the use of stable and reproducible SERS substrates, like the ordered NP structures, in combination with advanced multivariate analysis can be an effective approach for LTG quantification in biological samples.

It is evident that the development of alternative, straightforward, cost-, and time-effective methods for LTG and other AEDs detections are highly required for the effectiveness of drug therapeutics in epilepsy. However, one of the main limitations of several published methods is the difficulty of their automation before implementation into clinical settings, where the sample pre-treatment and detection should ideally be integrated into a small device, e.g., a microfluidic system. This feature can be addressed in the case of our presented assay due to the advantage of using small uniform SERS substrates (4 × 4 mm), which are scalable and reproducible in fabrication, and they have been used for several quantitative analytical assays [19–21]. Moreover, implementing the small sensing substrates with a compact Raman spectrometer can considerably lower the volume of sample and reduce the number of steps for sample preparation and analysis. This improvement can be achieved by combining these features into a single and automated device, which will reduce even more the sampling steps and the need of highly skilled personnel. In addition, due to the low complexity of the presented assay, it can reduce extra costs and be implemented in routine settings for drug detection. Therefore, this assay can represent a step closer to point-of-need TDM in clinics.

## Conclusions

In this work, we developed a straightforward, less time-consuming, and sensitive label-free SERS assay for detecting and quantifying LTG in human serum. We showed that combining SPE-based sample pre-treatment with a SERS substrate immersion assay, carrying out the measurement with a compact, benchtop Raman system, and implementing a multivariate spectral data analysis (PLS-R method), allowed us to obtain a high accuracy LTG quantification in human serum, even in the presence of interference drugs. Although this assay is limited to reaching extremely low concentrations, our approach is good enough to cover the suggested LTG therapeutic reference range in clinics. Moreover, the use of the small, reproducible, and stable Au SERS substrate in this work, combined with a compact Raman system, can allow the development of compact assays and their integration in automated microfluidic platforms.

In summary, we showed that this sensing approach is more straightforward and faster (around 30 min) to perform than conventional LC/MS or immunoassay techniques (hours to days), and these advantages can facilitate the use of this assay, with a high potential to be automated in a compact device that can be place in routine clinical settings, as an alternative analytical TDM method for LTG. Moreover, due to the versatility of this approach, this work can open the development of new assays for various AEDs, where point-of-need TDM is relevant to improve patient dose adjustment and healthcare.

### Supplementary Information

Below is the link to the electronic supplementary material.

Supplementary file1 (DOCX 2628 KB)
