# Supplementary material for: Label-free SERS assay combined with multivariate spectral data analysis for lamotrigine quantification in human serum
Source: Mikrochim Acta. 2023 Dec 1;190(12):495. doi: 10.1007/s00604-023-06085-3 (PMC10689517; doi:10.1007/s00604-023-06085-3)
Supplement: Supplementary file 1 — Supplementary file1 (DOCX 2628 KB) [file 604_2023_6085_MOESM1_ESM.docx]

**Electronic Supporting Material**

**Label-free SERS assay combined with multivariate spectral data analysis for lamotrigine quantification in human serum**

**Isidro Badillo-Ramírez*^1,2^, Selina A. J. Janssen^3^, Gohar Soufi^1,2^, Roman Slipets^1,2^ , Kinga Zór^1,2^ and Anja Boisen^1,2^**

^1^ Center for Intelligent Drug Delivery and Sensing Using Microcontainers and Nanomechanics (IDUN), Department of Health Technology, Technical University of Denmark, Kgs. Lyngby, 2800, Denmark.

^2^ BioInnovation Institute Foundation, Copenhagen N, 2200, Denmark.

^3^ Molecular Biosensing for Medical Diagnostics (MBx), Department of Biomedical Engineering. Eindhoven University of Technology, 5600 MB Eindhoven, The Netherlands.

**Methods:**

**M1. Detailed Au NP SERS substrate fabrication:**

The Si nanopillars (Si NP) were first fabricated by etching a polished 4 in. silicon wafer with a reactive ion etching (RIE) process, applying an SF_6_|O_2_ gas mixture for 4 min, obtaining vertically Si nanopillar structures (∼ 50 nm in width, ∼ 400 nm in height, and a Si NP density between 20 and 25 NP/μm^2^). Then the formed Si NP structures were exposed to O_2_ plasma for 1 min to clean the surface (MESC Multiplex ICP, STS, Morgan Hill, CA, USA). Next, a 225 nm thick Au metal film was deposited on the Si NP, obtaining mushroom-like structures. The Au coating was performed with a thermal evaporator (NANO36, The Kurt J. Lesker Company, PA, USA). Surface characterization of Au NP substrates was performed with scanning electron microscopy (SEM) using Zeiss Supra VP 40 (Jena, Germany).

**M2. A syringe filter holder (μ-SPE-SFH) fabrication:**

The μ-SPE-SFH was fabricated employing Oasis HLB as a sorbent. The LTG extraction conditions in SPE were based on a previously reported procedure by *Shah et al.* [1]. HLB is a universal polymeric reversed-phase resin consisting of N-vinylpyrrolidone (hydrophilic) and divinylbenzene (lipophilic) monomers, ideally used for the extraction of acidic, basic and neutral compounds [2]. Miniaturized cartridges with HLB sorbent were prepared by filling a syringe filter (25 mm, sterile, 0.2, Labsolute) with 1 mL of preconditioned sorbent in MeOH, which was prepared by placing 65 mg of HLB in 1 mL of MeOH for 24 h. Then, the syringe filter was washed with 1 mL of Milli-Q water.

**M3. Univariate and multivariate spectral data analysis for LTG quantification.**

Based on univariate and multivariate spectral data analysis, two approaches were used to build independent calibration plots for LTG quantification:

*a) Univariate spectral analysis. The area under the peak*

The band area value of the spectral position between 1300 and 1390 cm−1 was used to build a calibration plot. The calibration plots were obtained in PBS, commercial serum, centrifugal filtered serum, and serum collected after SPE, by plotting the average band area at position 1356 cm-1 versus the LTG concentrations. The limit of detection (LoD) was calculated based on the formula: LoD = Sblank + 3 · σblank, where Sblank represents the signal of the blank sample without LTG, and σblank is the sample standard deviation of the blank samples. The limit of quantification (LoQ) was calculated employing the three times LoD (3・LoD). For statistical analysis, three different SERS chips (n=3) were measured for each LTG concentration under identical experimental conditions. All data were plotted and fitted using linear and/or sigmoidal fittings in Origin (2020, Origin Lab Corporation, Northampton, MA, USA).

*b) Multivariate spectral analysis. The partial least-square regression (PLS-R) model*

A multivariate spectral calibration method was implemented to determine the analytical parameters for LTG quantification. The PLS-R model was selected with the aim to improve the modeling speed of spectral analysis while preserving the original data. A PLS-R model generates a linear equation to correlate the variations of the spectral data, the X variable, to a series of target concentrations, the Y variable. It can perform a full spectrum analysis and provides chemical information considering most of the entire vibrational modes. Quantitative prediction of LTG in complex samples with competitive molecules was performed after building a linear regression. The data matrix consisted of 18 rows (6 concentrations × 3 replicates) and 331 columns (band intensities). To evaluate the prediction of the model, 6 points of data set were kept outside to build the prediction set with three different methods: (i) randomly, (ii) leave out one replication from each concentration out and (iii) three points randomly plus three points from one concentration (18 µM) which was only utilized in the prediction set. The PLS-R model was performed using a PLS toolbox (Eigenvector Research) in Matlab (2021b, MathWorks, Natick, MA, USA). The optimal number of latent variables (LVs), equal to 3, was selected according to a commitment between the lowest root mean squared error cross-validation (RMSECV) and the lowest number of LVs while considering the minimum values of the root-mean-square error of calibration (RMSEC) and cross-validation (RMSECV) [3] . The prediction ability of the PLS-R model was estimated based on the RMSEC, RMSECV, root mean square error of prediction (RMSEP), the percent of prediction error, as well as the correlation coefficient (R^2^) between the actual concentrations of LTG and the predicted values, hence the variance percentage in the Y variable, which is explained by the X variable. The R2, the RMSEC, the RMSECV, and the bias between actual and predicted LTG concentrations were considered in the evaluation of the quality of the calibration model. Furthermore, quantification parameters such as sensitivity, LoD, LoQ, selectivity, and linear ranges were calculated and reported for the PLS-R model. The LoD and LoQ were calculated with Multivariate calibration 1 (MVC1), an integrated chemometric toolbox for Matlab.

**M4. Efficiency calculation of serum cleaning methods**

*a) Centrifugal filtration*

The efficiency of the centrifugal filtration method was calculated by comparing the band areas (between 1300 and 1390 cm^-1^) for the serum samples spiked with LTG before filtration (SbF) with the band areas for the blank serum samples spiked with LTG after filtration (SaF). The efficiency for each concentration was calculated by dividing SbF by SaF multiplied by 100%. The filtration efficiency is reported as the average efficiency, considering all the measured concentrations. The relative standard deviation (RSD) was determined by dividing the average filtration efficiency by the sample standard deviation of the calculated filtration efficiencies.

*b) SPE*

The extraction efficiency (EE) of the SPE procedure was determined by comparing the band areas (between 1300 and 1390 cm^-1^) for the serum samples spiked with LTG before SPE (SbSPE) with the band areas for the blank serum samples spiked with LTG after SPE (SaSPE). The EE for each concentration was calculated by dividing SbSPE by SaSPE multiplied by 0.5 and 100%. The 0.5 factor was applied to correct the up concentration in the SPE method since the starting sample volume was 1 mL, and the elution volume was 500 μL. The EE of the SPE procedure is reported as the average EE, considering only the concentrations of 10 μM and higher. The RSD was calculated by dividing the average EE by the sample standard deviation of the calculated EEs.

**
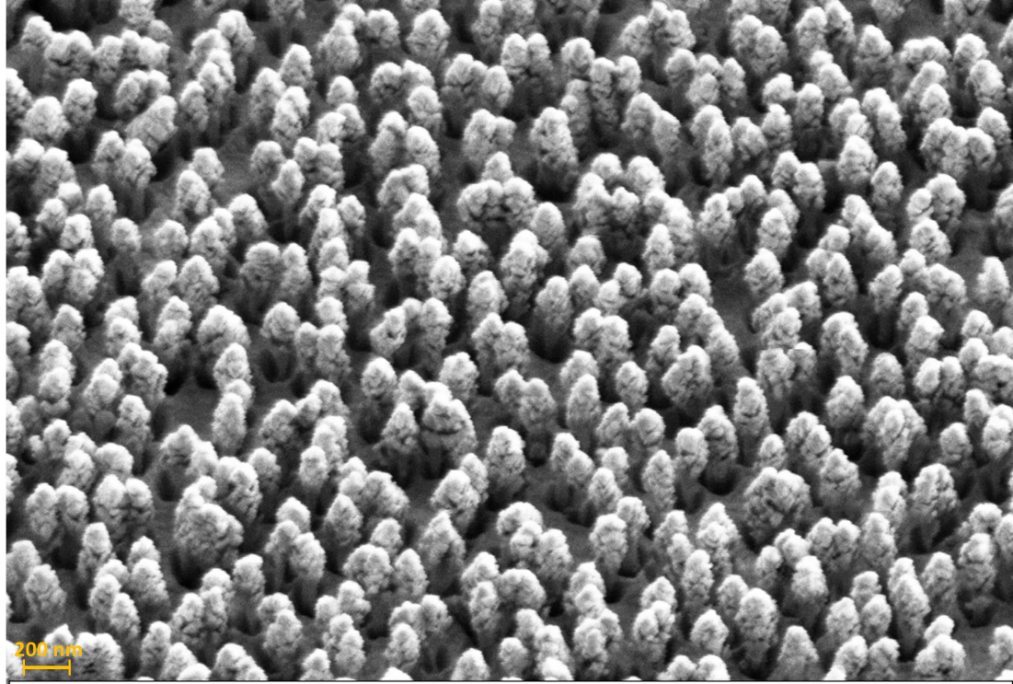
**

**Figure S1.** SEM image characterization of the fabricated Au NP structures in the SERS chip employed for LTG assay development. Ordered NP mushroom-like structures were obtained with ∼ 50 nm in width, ∼ 400 nm in height, and a Si NP density between 20 and 25 NP/μm^2^. Reproducible conditions with previous reported NP substrates [4–6].


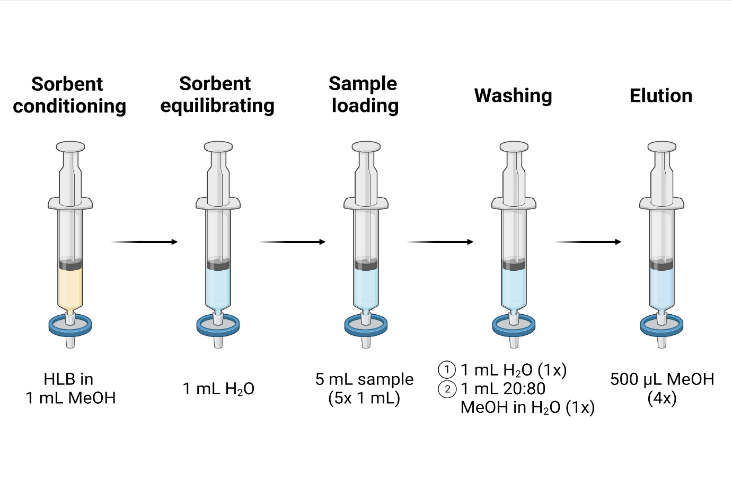


**Figure S2**. Schematic representation of the in-house developed SPE method (μ-SPE-SFH) for LTG separation from human serum. Sorbent conditioning, HLB sorbent is conditioned overnight in 1 mL MeOH. Sorbent equilibrating by passing 1 mL of Milli-Q water on the conditioning sorbent. Sample loading is performed by passing five times of 1 mL of spiked LTG in serum. Washing is carried out in two steps, first by passing 1 mL of Milli-Q water and then 1 mL of a mixture of MeOH in Milli-Q water (20:80). Elution is performed by passing for times of 500 µL of MeOH.

**Table S1.** Raman and SERS band assignments of LTG

| **Wavenumber (cm^-1^)** | | **Band assignments** [7–9] |
| --- | --- | --- |
| **Raman** | **SERS** |  |
| 1587 | 1596 | C-C arom. str. /NH_2_ sci. |
| 1559 | - | C-C str. /C=N str. |
| 1467 | 1467 | CH_2_ def. |
| 1434 | - | C- C str. / CH_2_ def. |
| 1326 | 1356 | CNC str. / C=N str. /C-H bend. |
| 1147 | 1158 | Arom. Breathing / NH_2_ sci. |
| 1057 | 1058 | Arom. C-H bend / NH_2_ rock. |
| 796 | 810 | CNC str. /NH_2_ rock. |
| 764 | - | CNC def. /C-C-C bend. |
| 617 | 610 | C-Cl str. / C-C-C bend. |
| 574 | - | C-Cl str. /NH_2_ twis. |
| 547 | - | C-Cl str./ NH_2_ def. |
| 476 | 482 | NH_2_ rock. |

**Abbreviations**: Arom., aromatic; bend., bending; def., deformation; rock., rocking; twis., twisting; sci., scissoring; str., stretching.

**a)**

**b)**


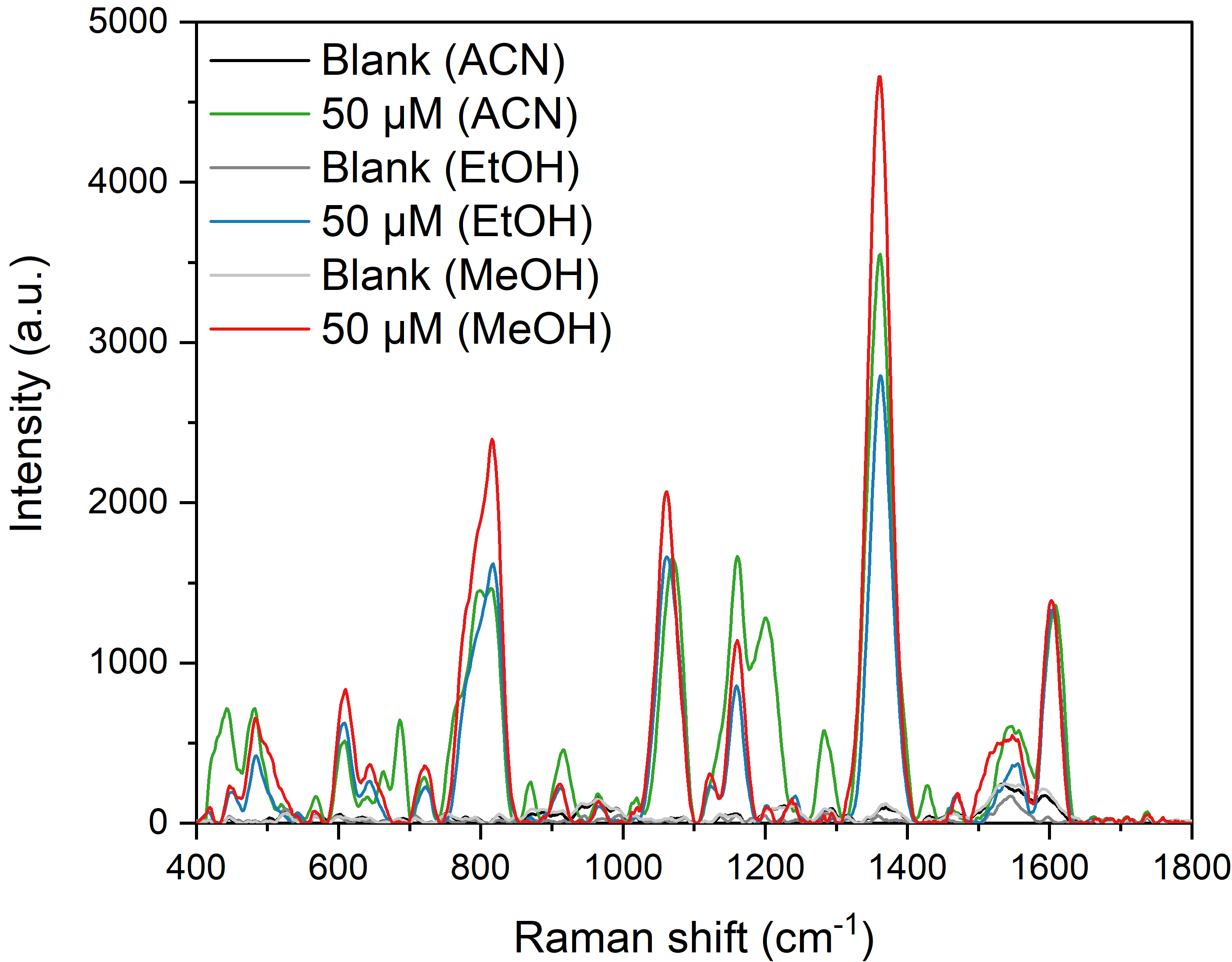

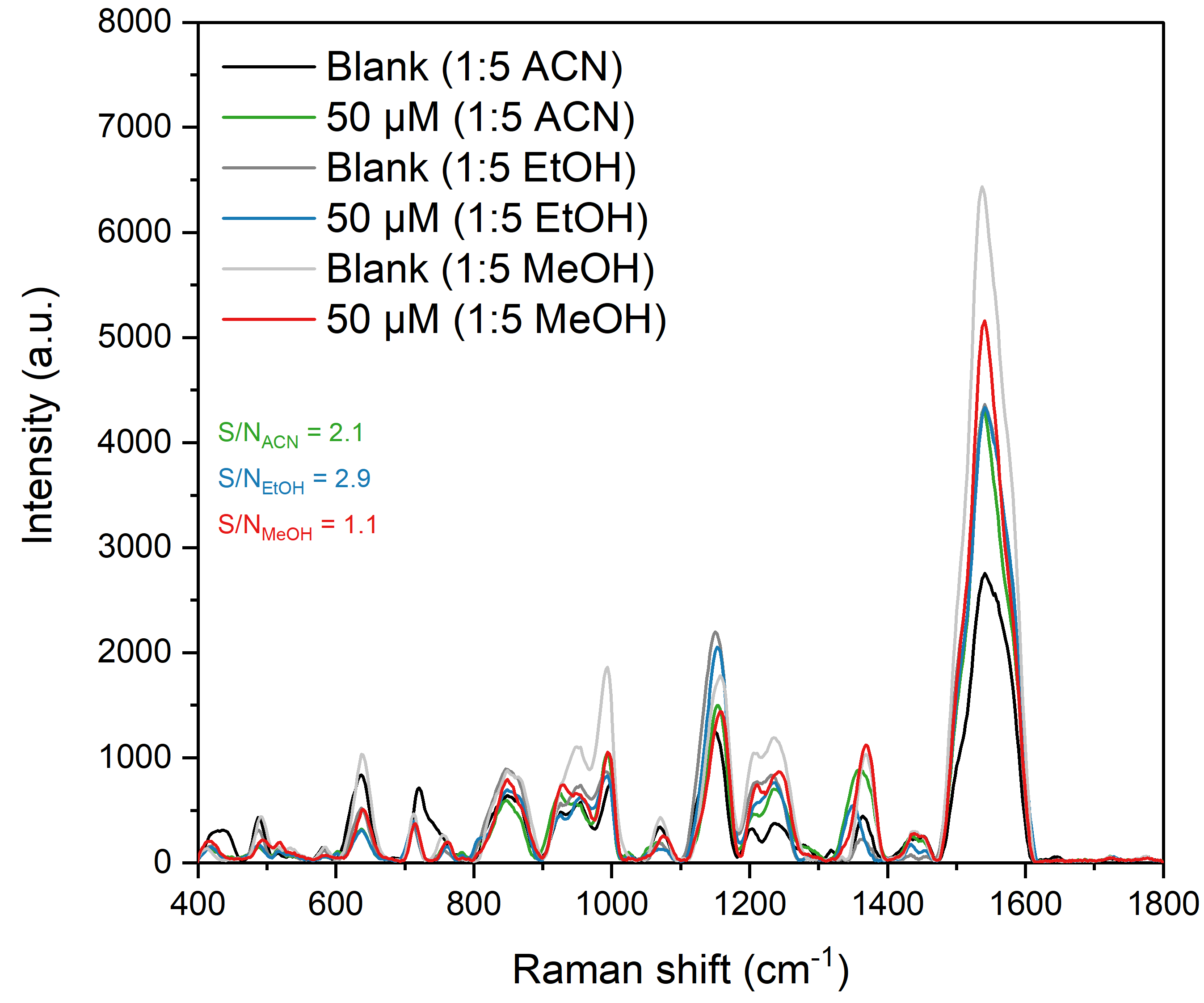


**Figure S3.** SERS-based sensing assay optimization conditions for LTG detection in PBS and serum. a) Comparative SERS spectra of LTG (50 µM) spiked in PBS (pH 7.4) and the blank after the mixture with different organic solvents (acetonitrile, ethanol, and methanol) in a 1:1 ratio. The comparison of the main LTG band intensity at 1356 cm^-1^ showed that MeOH was the ideal solvent for LTG migration on the SERS chip. b) Comparative SERS spectra of LTG (50 µM) spiked in human serum and in serum without LTG (blank) after the protein precipitation with different organic solvents (acetonitrile, ethanol, and methanol) in a 1:5 ratio. The highest S/N ratio (calculated according to the band area centered around 1356 cm^−1^) was obtained in a 1:5 ratio in EtOH. However, different mix rations with EtOH (1:5, 1:7 and 1:9) were evaluated for better protein precipitation, showing the 1:7 ratio with the highest S/N ratio; this was selected to build the calibration curve.

*
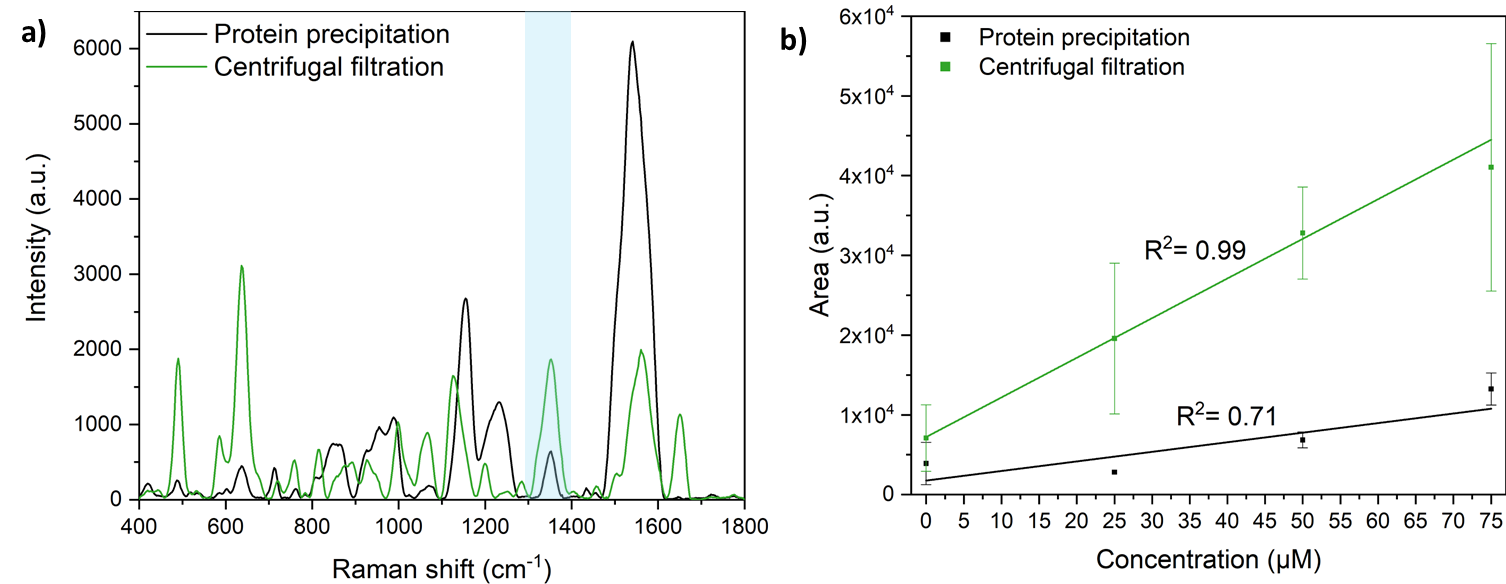
*

**Figure S4.** LTG separation with serum sample pre-treatment: a) Comparison of SERS spectra profile of LTG [50 μM] collected after protein precipitation and after centrifugal filtration; b) Comparative calibration curves of LTG with both separation methods, based on the peak area of the band at 1356 cm^-1^.

*
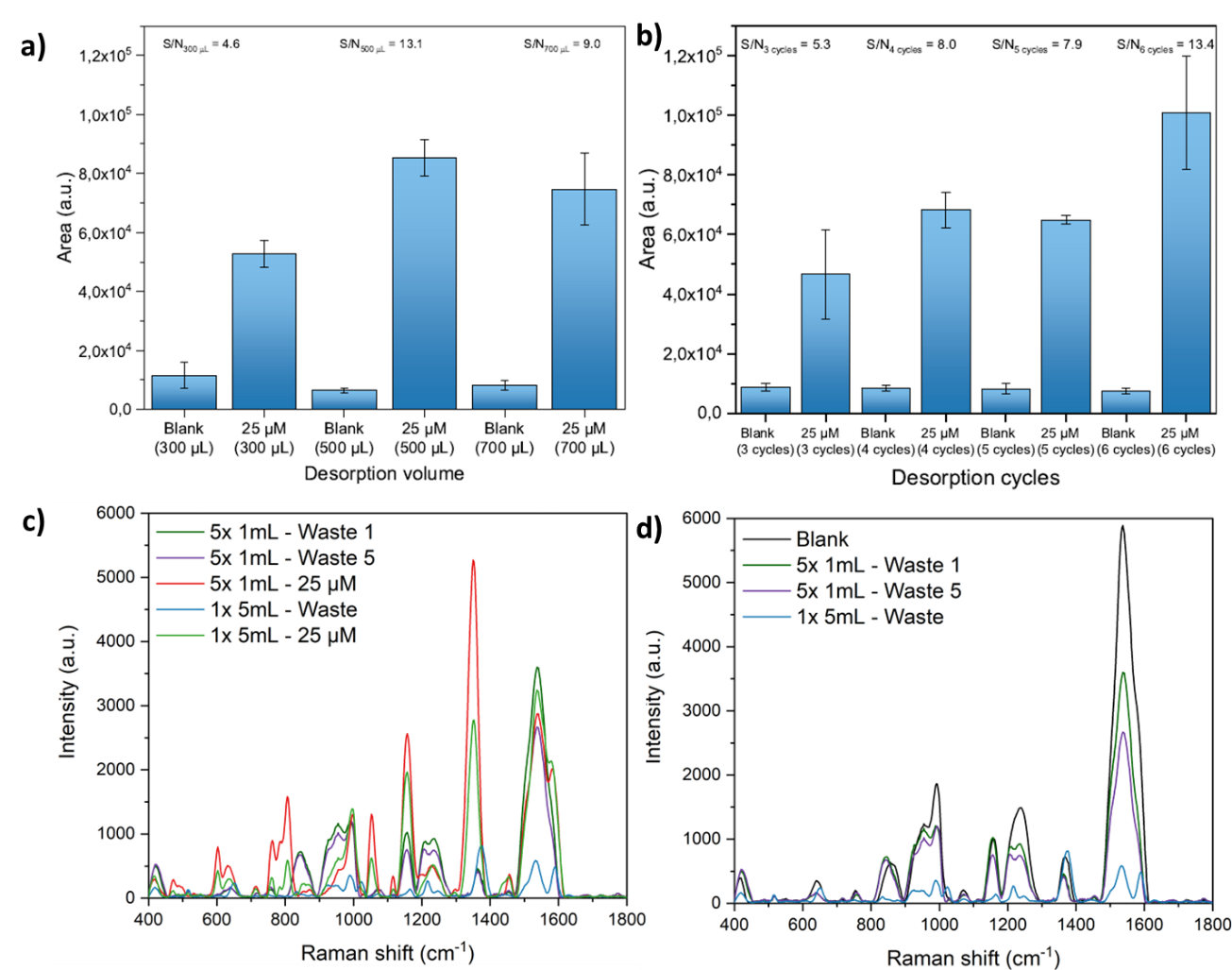
*

**Figure S5.** Optimized parameters for the SPE method. A) Selection of the amount of desorption volume with MeOH for LTG (25 μM) spiked in serum and after the SPE method; histograms showing the band areas (in the 1300-1390 cm^−1^ region) of blank and LTG for different MeOH desorption volumes, i.e., 300, 500, and 700 μL. The highest S/N ratio was obtained for 500 μL. B) Comparison of the band area values of blank and 25 μM LTG at different desorption cycles, i.e., 3, 4, 5, and 6, employing 500 μL of MeOH. A number of 4 cycles was selected due to the high S/N value and low standard deviation. C) Comparative spectra of LTG (25 μM) in serum after NPAS and SPE when the 5 mL sample is passed through the syringe filter in one step (1× 5 mL) and five steps of 1 mL (5× 1 mL). The solution that is passed through the filter called ’Waste’ is also analyzed, showing no LTG remnants. D) Comparison between the SERS spectra of the ’waste’ samples and the blank (serum without LTG spiked), showing no LTG remnants after the complete SPE method.

*
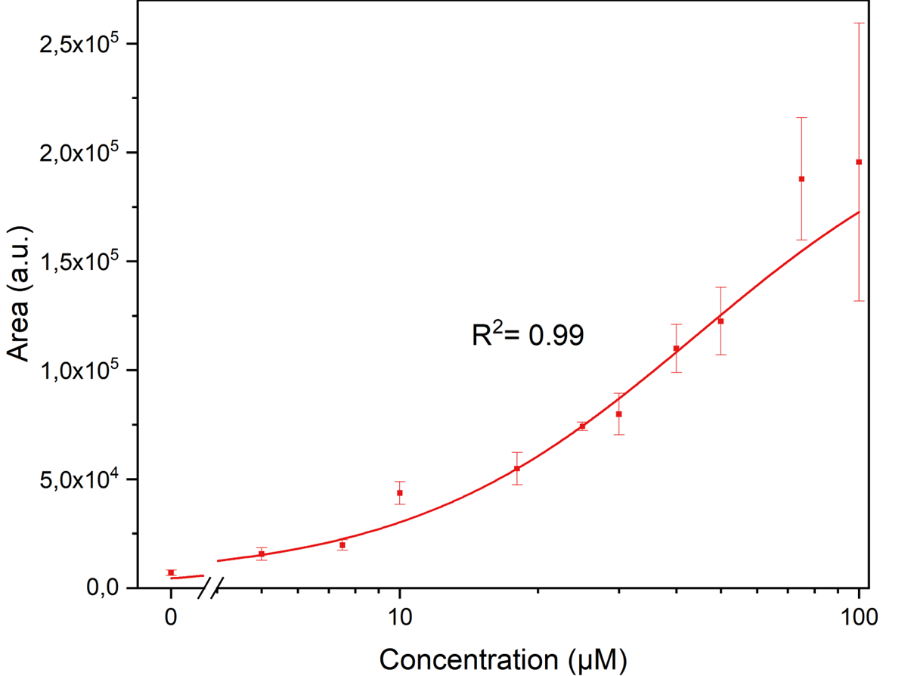
*

**Figure S6.** Calibration curve of LTG in the range from 5 to 100 µM with logarithmic curve fitting (sigmoidal fitting Hill equation) after combining the SPE method with the SERS-based sensing assay analysis.


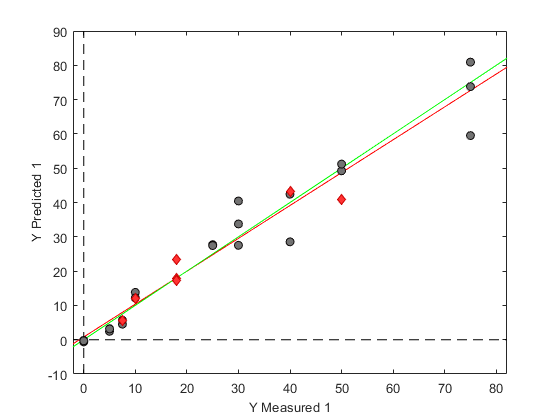

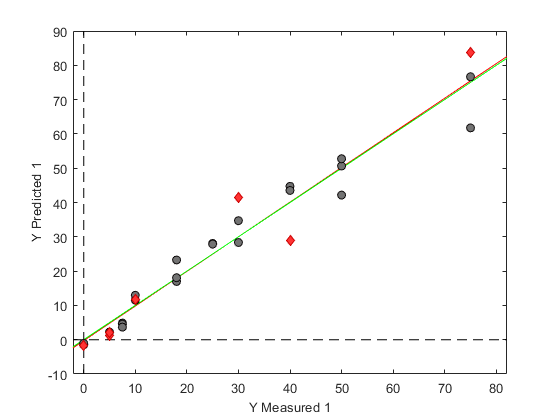


**a)**

**b)**

**Figure S7.** Calibration plots after the PLS-R analysis showing the effect of different prediction sets. The calibration set (black dots) and the prediction set (red dots) for the PLS-R model employing three LV and leave-one-out as cross-validation method. a) prediction set consisting of the 18 µM value which does not exist in the calibration set; and b) prediction set was chosen randomly, and from each concentration at least we have one point in the calibration set. Both show acceptable predictions as described in Table S2.

**Table S2.** Comparison of the different types of cross-validation methods and data set for predictive analysis employed in the PLS-R model for LTG quantification.

| ***Different types of cross-validation*** | ***Different data set for prediction*** |
| --- | --- |
| Cross validation: random samples w/ 10 splits  **RMSECV: 5.60314**  **R^2^ CV: 0.9457** | Without having 18 µM in the training set  **RMSEP: 4.55157**  **R^2^ Pred: 0.9299** |
| Cross validation: leave one out  **RMSECV: 5.50038**  **R^2^ CV: 0.9472** | Random selection of prediction  **RMSEP: 7.09241**  **R^2^ Pred: 0.9477** |

**Table S3.** Comparison of the LTG predicted concentration values by the PLS-R model versus the real LTG concentration in the sample.

| *Real LTG concentration (µM)* | *LTG predicted concentration (µM)* |
| --- | --- |
| *0* | *-0.86* |
| *5* | *2.63* |
| *7.5* | *5.27* |
| *10* | *12.19* |
| *18* | *23.98* |
| *25* | *28.52* |
| *30* | *28.85* |
| *40* | *39.02* |
| *50* | *53.51* |
| *75* | *84.78* |

*
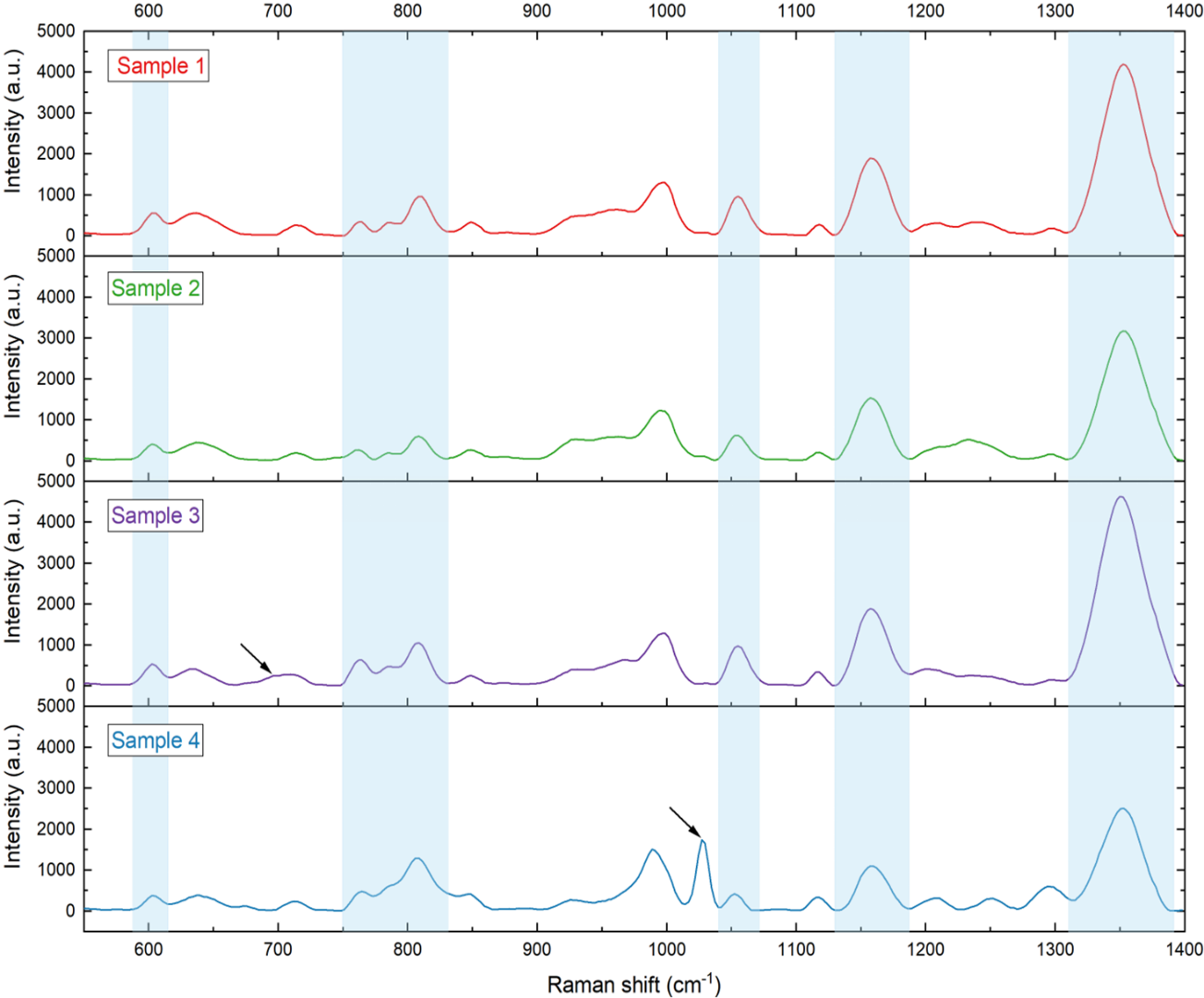
*

**Figure S8**. Average spectra profile of highly complex serum samples containing LTG (sample content and concentrations are indicated in Table 2) after combining SPE and SERS-based sensing assay methods for LTG quantification with the developed calibration models. Blue highlighted regions correspond to identified characteristic bands of LTG. Arrows indicate the characteristic bands of additional compounds in the prepared samples: 689 cm^-1^ and 1034 cm^-1^ for MTX and IMA in samples 3 and 4, respectively [5][10].

**References**

[1] H.J. Shah, G. Subbaiah, D.M. Patel, B.N. Suhagia, C.N. Patel, Rapid quantification of lamotrigine in human plasma by two LC systems connected with tandem MS, J. Chromatogr. Sci. 48 (2010) 375–381. https://doi.org/10.1093/chromsci/48.5.375.

[2] N.C. Dias, C.F. Poole, Mechanistic study of the sorption properties of Oasis® HLB and its use in solid-phase extraction, Chromatographia. 56 (2002) 269–275. https://doi.org/10.1007/BF02491931.

[3] K. Kachrimanis, D.E. Braun, U.J. Griesser, Quantitative analysis of paracetamol polymorphs in powder mixtures by FT-Raman spectroscopy and PLS regression, J. Pharm. Biomed. Anal. 43 (2007) 407–412. https://doi.org/10.1016/j.jpba.2006.07.032.

[4] O. Durucan, K. Wu, M. Viehrig, T. Rindzevicius, A. Boisen, Nanopillar-Assisted SERS Chromatography, ACS Sensors. 3 (2018) 2592–2598. https://doi.org/10.1021/acssensors.8b00887.

[5] Y. Göksel, K. Zor, T. Rindzevicius, B.E. Thorhauge Als-Nielsen, K. Schmiegelow, A. Boisen, Quantification of Methotrexate in Human Serum Using Surface-Enhanced Raman Scattering—Toward Therapeutic Drug Monitoring, ACS Sensors. 2 (2021). https://doi.org/10.1021/acssensors.1c00643.

[6] G. Soufi, E. Dumont, Y. Göksel, R. Slipets, R.A. Raja, K. Schmiegelow, H. Bagheri, A. Boisen, K. Zor, Discrimination and quantification of methotrexate in the presence of its metabolites in patient serum using SERS mapping, assisted by multivariate spectral data analysis, Biosens. Bioelectron. X. (2023) 100382.

[7] T.R. GR Ramkumaar, Structural and Qualitative Analysis of Lamotrigine, Int. J. Neurorehabilitation. 01 (2014) 2–5. https://doi.org/10.4172/2376-0281.1000135.

[8] G. Socrates, Infrared and Raman characteristic group frequencies: tables and charts, John Wiley & Sons, 2004.

[9] T. Ramya, S. Gunasekaran, G.R. Ramkumaar, Density functional theory, restricted hartree - Fock simulations and FTIR, FT-Raman and UV-Vis spectroscopic studies on lamotrigine, Spectrochim. Acta - Part A Mol. Biomol. Spectrosc. 114 (2013) 277–283. https://doi.org/10.1016/j.saa.2013.05.057.

[10] S. Fornasaro, A. Bonifacio, E. Marangon, M. Buzzo, G. Toffoli, T. Rindzevicius, M.S. Schmidt, V. Sergo, Label-Free Quantification of Anticancer Drug Imatinib in Human Plasma with Surface Enhanced Raman Spectroscopy, Anal. Chem. 90 (2018) 12670–12677. https://doi.org/10.1021/acs.analchem.8b02901.
